# A lack of nurse autonomy impacts population health when compared to physician care: an ecological study

**DOI:** 10.1038/s41598-023-38945-6

**Published:** 2023-07-25

**Authors:** Wenpeng You, Lynette Cusack, Frank Donnelly

**Affiliations:** 1 Adelaide Nursing School, The University of Adelaide, Adelaide, Australia; 2 Heart and Lung, Royal Adelaide Hospital, Adelaide, Australia; 3 Adelaide Medical School, The University of Adelaide, Adelaide, SA 5005 Australia

**Keywords:** Health policy, Health services, Medical ethics, Public health

## Abstract

This study highlights that the contribution of nursing is secondary to physicians in overall population health (indexed with life expectancy at birth, e_(0)_). Scatter plots, bivariate correlation and partial correlation models were performed to analyse the correlations between e_(0)_ and physician healthcare and nursing healthcare respectively. Affluence, urbanization and obesity were incorporated as the potential confounders. The Fisher’s r-to-z transformation was conducted for comparing the correlations. Multiple linear regression analyses were implemented for modelling that physicians’ contributions to e_(0)_ explain nurses’. Nursing healthcare correlated to e_(0)_ significantly less strongly than physician healthcare in simple regressions. Nursing healthcare was in weak or negligible correlation to e_(0)_ when physician healthcare was controlled individually or together with the three confounders. Physician healthcare remains significantly correlational to e_(0)_ when nursing healthcare alone was controlled or when the three confounders were controlled. Linear regression revealed that nursing healthcare was a significant predictor for e_(0)_ when physician healthcare was “not added” for modelling, but this predicting role became negligible when physician healthcare was “added”. Our study findings suggested that nurses still work under the direction of physicians due to lack of autonomy. Without correction, health services will continue to transmit the invisibility of nursing healthcare from one generation of nurses to another.

## Introduction

Life expectancy at birth (e_(0)_) integrates mortality patterns across all age groups^1^ and therefore is defined as how many years a newborn can expect to live, on average, if current mortality rates at different developmental stages do not change^1–3^. In epidemiology studies, e_(0)_ has been commonly considered as the statistical measure of overall population health^4^. The limited available data published by the World Bank data shows that, worldwide, e_(0)_ was 72.75 years, a 20 year improvement from 52.58 years in 1960^5^. The rapid increase of e_(0)_ has been attributable to epidemiological transitions which have remarkably improved human health^6^. For example, morbidity rate decrease across the human life span.﻿ Nurses and physicians as the two major practitioner groups in the healthcare workforce have contributed significantly to reducing mortality and morbidity rates to improve overall population health leading to the extension of e_(0)_^7–10^.

Nursing has been a female dominated profession^11,12^. Historically, nurses were considered as handmaidens or subordinates to a male-dominated medical profession. This doctrine established principles that required nurses to work under the supervision and direction of medical staff. It is commonly accepted that modern nursing started in mid-1900’s, an outcome of Nightingale’s Environmental Theory published in 1859. From that time, modern nursing has continued to strive for autonomy and recognition as a discipline equally important to medicine because both provide complimentary elements of healthcare^13,14^. This recognition, however, from the perspective of sociology, psychology and nursing history has consistently revealed that where there is a hierarchical physician- nurse relationship and there is often less effective team collaboration when nurses cannot exercise autonomy^15^ within their scope of practice. This ongoing hierarchical relationship contributes to nurses’ tension and stress which has been a contributing factor for nurses’ job dissatisfaction, burnout and attrition^16,17^.

Assuming that the role and responsibilities of both nurses and physicians across different populations are reasonably similar and the differences are negligible, nurse and physician densities are reasonable measures of nursing and physician healthcare respectively^7,8^. Based on this assumption, two previous studies have advanced that the overall roles and functions of both nurses and physicians for providing healthcare have been significantly associated with overall population health indexed with life expectancy at birth (e_(0)_)^7,8^. Using the same sources of data, this study recalculated and compared the two different levels of healthcare effects on overall population health worldwide. Firstly, we recalculated the correlations of physicians and nursing healthcare to overall population health respectively, and then compared the two correlations in multiple data analysis models. Finally, we analysed statistical explanatory effects of one another in terms of their contributions to overall population health. These statistical analysis results were applied to support our hypothesis that, globally, nursing healthcare promotes overall population health (e_(0)_) depending on the contribution of physicians.

### Study materials

All six variables included in this study were extracted from the database of the World Bank:

The two independent variables, nursing healthcare level and physician healthcare level are measured as healthcare professional densities, i.e. the number of nurses and physicians per 1000 population respectively^18,19^. Assuming that the role and responsibilities of both nurses and physicians across populations are similar and the differences are negligible, nurse and physician densities are reasonable measures of the levels of nursing and physician healthcare respectively^7,8^.

Nurse healthcare level is interchangeably written as nurses and nursing healthcare, and similarly physician healthcare level is interchangeably written as physicians or medical healthcare respectively in this article. To reduce possible errors when the World Bank collected and integrated the data, the numbers of nurses and physicians per 1000 population in each country between 2014 and 2018 were averaged respectively.

The dependent variable, life expectancy at birth (e_(0)_), reflects the overall mortality level of a population, and has been the most commonly used measure to describe overall population health^5^. In this manuscript, e_(0)_ and overall population health are interchangeable.

Gross Domestic Product (GDP), urban advantage and obesity have been postulated as the major factors influencing overall population health^8^. Therefore, they were potential confounders when we analysed and compared the relationships between e_(0)_ and physician and nurses healthcare levels respectively.

4. GDP purchasing power parity (GDP PPP) measures life quality and wellbeing of individuals at population level, which has constantly extended e_(0)_ of each individual country^20–22^.

5. Obesity increases the risk for developing health challenges which might lead to early mortality, and subsequently reduction of e_(0)_^23,24^.

6. Urbanization represents the level of advantages for urban residents to access better healthcare services provided by physicians and nurses and healthcare education opportunities^9,25^.

In total, a list of 215 countries with GDP PPP was downloaded and then the other five variables were matched with this list. Some populations did not have all the data for all the five variables. Therefore, the numbers of populations included in our data analysis models may differ as such. Politically speaking, not all the populations from which the international organisations collected data should be called “country”. For instance, the data from Hong Kong and Macau are included in this study, but neither of them has a sovereign title. For the purposes of this paper a territory with or without sovereign is called a “country”, and it is interchangeable with the population.

### Ethical approval

All the population level data for this study were freely downloaded from the official website of the World Bank^26^. There are no individual people or communities who are identifiable in the study. The health information involved in this study is not traceable to any individual, their family and their community. Ethical clearance for conducting this study was obtained from the Office of Research Ethics, Compliance and Integrity (ORECI) of the University of Adelaide (Ethics Approval Number: 36289).

### Data analysis models

The relationships between e_(0)_ and nurse density and physician density were calculated and compared in four common data analysis models, which were adopted in previous studies^7,8,27,28^.

1. Scatter plots were conducted with the raw data in Microsoft Excel 2016^®^.

This does not only allow us to check the data quality, for instance checking if there is any outlier, but also allows us to calculate and visualize the strengths and directions of correlation of e_(0)_ with nursing healthcare and physician healthcare respectively.

The square roots of the two R square values were obtained for comparing the correlations of e_(0)_ to nursing healthcare and physician healthcare.

2. Pearson’s r and nonparametric correlations were performed for evaluating the strengths and directions of the bivariate correlations between all the six variables. The correlations between variables allow us to collate the data selection and inclusion of the potential confounding variables with previous studies.

3. Partial correlation of Pearson’s moment-product model were conducted to reveal the independent correlations between different pairs of variables. Firstly, we controlled for GDP PPP, obesity prevalence and urban advantages for calculating the correlations between e_(0)_ and nursing healthcare and physician healthcare respectively. This allows us to explore if and how much the potential competing effects of GDP PPP, obesity prevalence and urbanization make on the significantly correlations between e_(0)_ and nursing healthcare and physician healthcare. And then we alternated nursing healthcare and physician healthcare as a potential confounder, and controlled together with affluence, obesity and urbanization for examining the correlation between the one another’s correlation with e_(0)_. Further to this, we alternated each of the five variables (independent and confounders) as the predictor for assessing the correlation between e_(0)_ and each of the five variables while all the rest of four variables were considered as the potential confounders.

Finally, we alternated each of the five individual variables (physician healthcare, nursing healthcare, affluence, obesity and urban advantages) as the potential competing variable to reveal the correlations of e_(0)_ to the other four individual variables respectively.

The Fisher’s r-to-z transformation was conducted to demonstrate that e_(0_) was a significantly weaker correlation to nursing healthcare than to physician healthcare in the data analysis models, Pearson’s r nonparametric and partial correlation.

4. Standard multiple linear regression (enter) was performed to reveal the correlations between e_(0)_ and the predicting variables (nursing healthcare and physician healthcare). In order to reveal if and how much nursing healthcare can statistically explain the individual correlations of e_(0)_ to physicians, affluence, obesity and urban advantages, the enter multiple linear regression model was conducted to reveal the correlations of e_(0)_ to these individual variables when physicians was “not added” and “added” as an independent/predicting variable respectively. Subsequently, stepwise standard multiple linear regression model was conducted to select the predicting variable(s) which had the most significant effects on e_(0)_ when physician healthcare was “not added” and “added” as an independent/predicting variable respectively.

We alternated nursing healthcare and physician healthcare, and repeated the above linear regression models for observing if and how much nursing can statistically explain physician, GDP PPP, obesity prevalence and urbanization.

We log transformed the variables for increased homoscedasticity for data correlation analyses. SPSS IBM v. 28® was conducted for exploring the bivariate correlations (Pearson’s r and nonparametric), partial correlation and multiple linear regression analyses. The significance of the correlation was set at the 0.05, but the levels of 0.01 and 0.001 were reported as well. The analysis criteria of standard multiple linear regression (enter and stepwise) were kept at probability of F to enter ≤ 0.05 and probability of F to remove ≥ 0.10.

## Results

**Figure 1:**
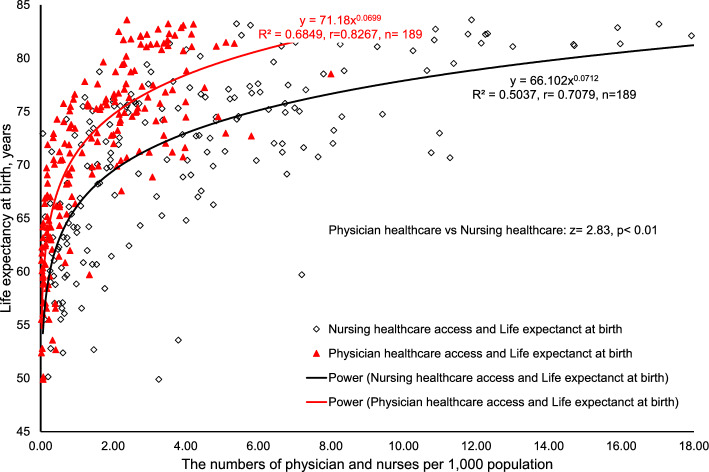
The relationships between life expectancy at birth and physician and nursing healthcare respectively.

The relationships revealed in the scatterplots between e_(0)_ and nursing healthcare and physician healthcare were both noted to be power with strong correlations (R^2^ = 0.5037, p < 0.001, n = 189 and R^2^ = 0.6849, p < 0.001, n = 189 respectively, Fig. 1). There was no major outlier in either nursing healthcare, physician healthcare or e_(0)_. Worldwide, nursing healthcare and physician healthcare explained 50.37% and 68.49% of e_(0)_ respectively. When the above two R^2^’s were calculated into coefficient r’s for revealing the significance of difference, it was found that nursing healthcare was in significantly weaker correlation to e_(0)_ than physician healthcare (z = − 2.83, p < 0.01).

The significant predicting effects of nursing healthcare and physician healthcare on e_(0)_ and their significant difference revealed in the scatterplots were consistent with the subsequent bivariate correlation analyses with log-transformed data.

Worldwide, both nursing healthcare and physician healthcare significantly correlated to e_(0)_ (r = 0.695 and r = 0.822, p < 0.001 in Pearson respectively). Fisher r-to-z transformation identifies that nursing healthcare was in significantly weaker correlation to e_(0)_ than physician healthcare (z = − 2.95, p < 0.01). Similarly, in non-parametric model, both nursing healthcare and physician healthcare significantly correlated to e_(0)_ as well (r = 0.721 and r = 0.807, p < 0.001 respectively). Fisher r-to-z transformation also identified that nursing healthcare was in significantly weaker correlation to e_(0)_ than physician healthcare (z = − 2.01, p < 0.05) (Table 1).

**Table 1:** Pearson (above diagonal) and non-parametric (below diagonal) correlation matrix for all variables.

	Nursing healthcare	Physician healthcare	Life expectancy at birth	GDP PPP	Urbanization	Obesity prevalence
Nursing healthcare	1.000	0.784***	0.695***	0.774***	0.547***	0.508***
Physician healthcare	0.810***	1.000	0.822***	0.839***	0.626***	0.500***
Life expectancy at birth	0.721***	0.807***	1.000	0.801***	0.528***	0.427***
GDP PPP	0.797***	0.831***	0.842***	1.000	0.720***	0.502***
Urbanization	0.600***	0.630***	0.620***	0.757***	1.000	0.546***
Obesity prevalence	0.465***	0.445***	0.437***	0.483***	0.584***	1.000

Fisher r-to-z transformation identifies life expectancy at birth is in significantly stronger correlation with physician healthcare than with nursing healthcare in Pearson r (z = 2.95, p < 0.01) and non-parametric (z = 2.01, p < 0.05) models.

Significance level: ***p˂ 0.001; n ranges between 176 and 194.

Data source and definition: Nursing healthcare, the number of nurses and midwives per 1,000 population (the Word Bank); Physician healthcare, the number of nurses and midwives per 1,000 population (the Word Bank); Life expectancy at birth, the average number of years that a newborn could expect to live (the World Bank); GDP PPP, the per capita purchasing power parity (PPP) value of all final goods and services produced within a territory in a given year (the Word Bank); Urbanization, the percentage of population living in urban area (the Word Bank); Obesity prevalence, the percentage of population with BMI ≥ 30 prevalence (WHO Global Health Observatory).

All the data were log-transformed for correlation analysis.

When the three potential confounders were controlled, both nursing and physician healthcare correlated to e_(0)_ significantly (r = 0.181, p < 0.05 and r = 0.471, p < 0.001 respectively). Fisher r-to-z transformation revealed that nursing healthcare was in significantly weaker correlation to e_(0)_ than physician healthcare (z = − 3.03, p < 0.01) (Table 2-1). When nursing healthcare and physician healthcare were alternated as the potential confounders together with GDP PPP, obesity and urbanization for exploring one another’s correlation to e_(0)_, nurses showed nil correlation to e_(0)_ (r = 0.001, p = 0.985), but physicians showed moderately strong correlation to e_(0)_ (r = 0.444, p < 0.001, Table 2-2).

**Table 2:** Partial correlation coefficients between life expectancy at birth and nursing healthcare and physician healthcare with different combinations of controlled variables.

Variables	2–1: Nursing healthcare and Physician healthcare to predict life expectancy at birth respectively while GDP PPP, obesity and urbanization were kept statistically constant						2–2: Nursing healthcare and Physician healthcare to predict life expectancy at birth respectively while one another, GDP PPP, obesity and urbanization were kept statistically constant						2–3. Nursing healthcare, Physician healthcare, GDP PPP, obesity and urbanization were alternated as the individual potential confounder for exploring the partial correlations between life expectancy at birth and the other four individual independent variables					
	Life expectancy at birth			Life expectancy at birth			Life expectancy at birth			Life expectancy at birth			Life expectancy at birth			Life expectancy at birth		
	r	p	df	r	p	df	r	p	df	r	p	df	r	p	df	r	p	df
Nursing healthcare	0.181	< 0.05	170	Not added			0.001	0.985	169	–	–	–	–	–	–	0.142	0.053	185
Physician healthcare	Not added			0.471	< 0.001	176	–	–	–	0.444	< 0.001	169	0.380	< 0.001	188	–	–	–
Obesity prevalence	–	–	–	–	–	–	–	–	–	–	–	–	0.590	< 0.001	180	0.032	0.670	180
GDP PPP	–	–	–	–	–	–	–	–	–	–	–	–	0.621	< 0.001	185	0.360	< 0.001	180
Urbanization	–	–	–	–	–	–	–	–	–	–	–	–	0.191	< 0.010	181	0.029	0.690	185

Fisher r-to-z transformation identifies that physician healthcare is in significantly stronger correlation to LEB than nursing healthcare (z = 3.03, p < 0.01).

– Controlled variable: All the data were log-transformed for correlation analysis.

Data source and definition: Nursing healthcare, the number of nurses and midwives per 1000 population (the Word Bank); Physician healthcare, the number of nurses and midwives per 1000 population (the Word Bank); Life expectancy at birth, the average number of years that a newborn could expect to live (the World Bank); GDP PPP, the per capita purchasing power parity (PPP) value of all final goods and services produced within a territory in a given year (the Word Bank); Urbanization, the percentage of population living in urban area (the Word Bank); Obesity prevalence, the percentage of population with BMI ≥ 30 prevalence (WHO Global Health Observatory).

When nursing healthcare, physician healthcare, GDP PPP, obesity and urbanization were individually controlled, physician healthcare is moderately strong and significant correlation with e_(0)_ independent of nursing healthcare (r = 0.621, p < 0.001, Table 2-3). However, when physician healthcare was kept constant, nursing healthcare only showed very weak and insignificant correlation to e_(0)_ (r = 0.142, p = 0.053, Table 2-3). These results suggested that nursing healthcare significantly contributes to e_(0)_, but the magnitude became insignificant when physician healthcare was also considered.

When physician healthcare was “not added” as one of the predicting variables, standard multiple linear regression (enter) analysis revealed that GDP PPP and nursing healthcare were the only two significant predicting variables for e_(0)_ (β = 0.698, p < 0.001 and β = 0.174, p < 0.05 respectively Table 3-1). Obesity and urbanization showed almost nil contribution to e_(0)_. Together with GDP PPP, physician healthcare was selected as a significant contribution to e_(0)_ (β = 0.527 and 0.426 respectively, p < 0.001) when physician healthcare was “added” as one of the predicting variables in the enter model. Nursing healthcare, obesity prevalence and urban advantages showed nearly nil correlation to e_(0)_ (Table 3-1).

**Table 3:** Multiple linear regression results to show predicting effects of independent variables and identify the significant predictors of life expectancy at birth.

Variable	Life expectancy at birth									
	3–1 Enter				3–2 Stepwise					
	Physician healthcare (not added)		Physician healthcare(added)		Physician healthcare (not added)			Physician healthcare (added)		
	Beta	Sig	Beta	Sig	Rank	Variable	Adjusted R^2^	Rank	Variable	Adjusted R^2^
Nursing healthcare	0.174	< 0.05	0.001	0.985	1	GDP PPP	0.627	1	Physician healthcare	0.668
GDP PPP	0.698	< 0.001	0.425	< 0.001	2	Nursing healthcare	0.638	2	GDP PPP	0.706
Obesity prevalence	0.012	0.839	− 0.002	0.966		Obesity prevalence	Insignificant		Nursing healthcare	Insignificant
Urbanization	− 0.062	0.385	− 0.099	0.121		Urbanization	Insignificant		Obesity prevalence	Insignificant
Physician healthcare	Not added		0.527	< 0.001		Physician healthcare	Not added		Urbanization	Insignificant

Variable	Life expectancy at birth									
	3–3 Enter				3–4 Stepwise					
	Nursing workforce (not added)		Nurse number (added)		Nursing workforce (not added)			Nursing workforce (added)		
	Beta	Sig	Beta	Sig	Rank	Variable	Adjusted R^2^	Rank	Variable	Adjusted R^2^
Physician healthcare	0.527	< 0.001	0.527	< 0.001	1	Physician healthcare	0.668	1	Physician healthcare	0.668
GDP PPP	0.426	< 0.001	0.001	0.985	2	GDP PPP	0.706	2	GDP PPP	0.706
Obesity prevalence	− 0.002	0.968	0.425	< 0.001		Obesity prevalence	Insignificant		Nursing healthcare	Insignificant
Urbanization	− 0.099	0.117	− 0.002	0.966		Urbanization	Insignificant		Obesity prevalence	Insignificant
Nursing healthcare	Not added		− 0.099	0.121		Nursing healthcare	Not added		Urbanization	Insignificant

Significance level: *p < 0.05; **p˂ 0.01; ***p˂ 0.001; All the data were log-transformed for correlation analysis.

Data source and definition: Nursing healthcare, the number of nurses and midwives per 1000 population (the Word Bank); Physician healthcare, the number of nurses and midwives per 1000 population (the Word Bank); Life expectancy at birth, the average number of years that a newborn could expect to live (the World Bank); GDP PPP, the per capita purchasing power parity (PPP) value of all final goods and services produced within a territory in a given year (the Word Bank); Urbanization, the percentage of population living in urban area (the Word Bank); Obesity prevalence, the percentage of population with BMI ≥ 30 prevalence (WHO Global Health Observatory).

Similarly, in the stepwise model, when physician healthcare was “not added” as one of the predictors, GDP PPP and nursing healthcare were the only two significant predictors for e_(0)_ (R^2^ = 0.627 and 0.638 respectively, Table 3-2). However, physician healthcare was selected as the most influential contributor to e_(0)_ (R^2^ = 0.668) when it was “added” as an independent/predicting variable. In this model, GDP PPP was placed as the second most influential predictor for e_(0)_ with the R^2^ increment of 0.038 (Table 3-2), and nursing healthcare was not selected as a significant predictor for e_(0)_. In this data analysis model (stepwise), totally, 70.60% of e_(0)_ was explained by nursing healthcare, physician healthcare, GDP PPP, obesity prevalence and urbanization (Table 3-2).

When nursing healthcare was “not added” as a predicting variable in linear regression enter model, physician healthcare and GDP PPP significantly correlated to e_(0)_ (β = 0.527 and β = 0.426, p < 0.001 respectively, Table 3-3). When nursing healthcare was “added”, this did not change the correlations between e_(0)_ and each of four predicting variables (physician healthcare, GDP PPP, obesity prevalence and urbanization). Similarly, in the two stepwise models, when nursing healthcare was “not added” and “added” in the analyses, physician healthcare and GDP PPP were the only two variables showing most influential predicting effects on e_(0)_, and the addition of nursing healthcare did not affect how much the e_(0)_ was explained (both 70.60%, Table 3-3 and -4).

These results appeared in linear regression models were consistent with those reported in Table 2. The statistical relationships reported in Tables 2 and 3 suggested that nursing healthcare significantly contributed to e_(0)_, which was dependent on physicians’ contribution.

## Discussion

The findings above illustrate that while nurses account for the bulk of the health care workforce their contribution to discrete indices such as life expectancy are difficult to identify. Our research findings confirm the overt recognition physicians receive in healthcare service delivery, when comparing physicians’ and nurses’ contributions to overall population health (measured with e_(0)_):

1. Nursing healthcare contributes to e_(0)_ significantly less than physician healthcare, and this significant disparity remains although we ruled out the competing effects of the common confounding factors associated with e_(0)_, such as economic affluence, urbanization and obesity.
2. Physician healthcare service may contribute to e_(0)_ independent of nursing healthcare service. However, nursing healthcare appears to play a very minor role in maintaining and improving e_(0)_, when physician’s contribution to e_(0)_ is controlled. This suggests that the nursing industry influences overall population healthcare dependent on physician workforce.
3. Physicians’ role may ‘statistically’ explain nurses’ responsibility for contributing to e_(0)_ when both physician healthcare and nursing healthcare, economic affluence, urbanization, and obesity were incorporated for analysing their relationships with e_(0)_. In contrast however nursing healthcare shows negligible explanation for physicians’ healthcare for maintaining and improving e_(0)_. This suggests that the nursing role and contribution to population health is hidden within the measures used to identify physicians’ contributions to overall population healthcare.

Fundamentally, the statistical role of nursing healthcare in promoting overall population health is dependent on physicians’ contribution. This may suggest that nursing industry lacks autonomy, which is defined as a nurse's ability to apply professional knowledge and experience to healthcare and make independent clinical decisions regarding patient care. With autonomy, nurses should rightfully expect equity in decision-making processes, a reciprocal exchange with physicians while developing and implementing the patient’s healthcare plan and a more refined set of metrics used to measure discrete nursing impact^29^. In determining the non-medical related aspects of patient’s care, nurses have an opportunity to establish autonomous principles of care. Although nursing has been striving for autonomy since 1845^30^, our study results highlight that the nursing industry is still not recognised as an independent contributor to overall population health. Studies examining the variations of the “physician- nurse game” continue to describe that medical teams largely direct nursing workforce in the delivery of physician measures of healthcare services^31–34^. Illustrated in the statistical relationships between variables, lack of autonomy in nursing industry can be evidenced with the phenomena which commonly appear in healthcare systems:

1. While noting that medicine education for physicians is typically longer than for other health professions^35^, nurses’ educational level and status usually affords less authority in healthcare contexts. The education disparity between physicians and nurses has led to imbalanced power between nurses and physicians which encourages physicians to take a role, which is authoritative to that of the nurse^36^. Worldwide, physicians tend to hold privileged positions of control and remuneration, they are often the legal determinant of decisions for patients’ healthcare^37^. This overt dominance may have been reflected in the statistically significant differences between the roles and functions of physicians and nurses identified in promoting overall population health in our study^38,39^.
2. In clinical settings, the roles and functions of nurses in promoting overall population health (e_(0)_) may not be viewed or documented as equally important as physicians by the healthcare providing facilities and the public. This inference has been supported by several studies which concluded that, in the last decades, although nurses and physicians have been collaborating in different ways, overall, physicians remain dominant in healthcare services^31–34^.

Nursing healthcare was in significant and moderate correlation to e_(0)_, but this relationship became very weak and insignificant when physicians were incorporated for both partial correlation and multiple linear correlation analyses. This may suggest that nursing industry lacks autonomy, which drives nurses to make nursing clinical healthcare decisions only based on physicians’ diagnosis and treatment plan. The contribution of nurses to e_(0)_ in this nurse-physician collaboration model may not allow the role of nursing to be significantly noticeable as nurses follow physicians healthcare decision, instead of exercising autonomy for nursing specific healthcare plans^40^. Nursing has come a long way since Nightingale, and nurses nowadays may have different expectations in their collaboration with their medical colleagues. If however, the medical profession still consider nurses as subservient this may lead to nurse-physician conflict, which in turn leads to job dissatisfaction and,^41,42^ therefore contribute to reducing the nursing workforce.

In addition to the potential for nurse-physician conflicts, nursing work is physically and mentally demanding^43–45^, as nurses are at the frontline of delivering increasing complex care to patients and their family, in fast paced health care services. These pressures on nurses with the added nurse-physician conflict only increases the risk of burnout and job dissatisfaction^44,46^, and eventually exit from a nursing career^44,45,47^. The professional impact of premature exits due to physician-nurse conflict and job dissatisfaction is that the nursing industry may not benefit from deep level of experience when compared to physicians. This may contribute to not only the significant disparity in healthcare provided by physicians and nurses, but also further reduce the opportunity to collect and influence measures of healthcare effects such as life expectancy^48,49^. The risk of a less experienced workforce is a vicious cycle where nurses with less confidence, skills and knowledge fail to assert their contribution to patient care issues. This also diminishes the opportunity for the development of advanced nursing practice and Nurse Practitioner roles.

Some studies showed that nurses and physicians might have similar positive patient outcomes in primary healthcare service settings^50–52^. However, this is significantly different from our study design which we compared the roles and responsibilities of nurses and physicians for promoting overall population health across primary, secondary and tertiary healthcare settings.

### Implications for nursing practice and health policy

The study findings may stimulate health authorities and the nursing industry to consider identifying other metrics to track impact. Health authorities should make nursing autonomy more explicit, supported and recognised. They also should provide continuous support to nursing industry for improving their autonomy through re-defining notions of autonomy, developing educational content^53^ and especially clearly defining and expanding scopes of nursing practice. Without continuous improvement in nursing autonomy, the risk of nurse-physician conflict remains and may further contribute to nurses’ burnout and job dissatisfaction. Nurses leaving the profession place further demands on healthcare systems, the loss of experienced nurses contributes to instability.

It is also important that, while nurses play a complementary role to physicians^54,55^, their contribution needs to be more visible when considering patients’ healthcare plan and patient satisfaction^56–58^. This can be resolved through implementing clear metrics for nursing interventions^59,60^.

### Limitation and strength

Firstly, this is an ecological study, and the results are dependent on the nature of the ecological fallacy. The population level correlational relationships revealed in our data analyses might not hold true at the individual level. However, considering the number and distribution of people involved in the study, this may not be achievable in the individual based study.

Secondly, the relationships revealed in this data analysis-based study are not causal, but correlational.

Thirdly, the data included in this study may be fairly crude and they may have some random errors when the population level data were collected and aggregated by the United Nations agencies. However, these data were collected and integrated in a more objective manner, which is different from how the data collect for the individual studies.

It is also noted that the total number of nurses and physicians represents over 70% of healthcare employment. While the valuable contribution of other allied healthcare professionals is acknowledged a lack of specific discipline data prevents their inclusion in our data analyses. Considering that health authorities prioritize employment of nurses and physicians due to limited finance budgets, the impact of other healthcare professionals on our data analysis results could be alleviated by controlling GDP PPP in the data analysis models.

Finally, there is high validity of the variables involved in this study. Physicians, nurses, GDP PPP, urbanization and obesity explain a majority of overall population health (R square incremented to 70.6%, Table 3). Other sources of data concerning other health care variables such as physical exercise, blood pressure, cholesterol are difficult to extract for population based studies. However, if such data were available, we might be able to demonstrate and compare more thoroughly the roles of nurses and physicians in determining life expectancy.

## Conclusions

Our study suggests that, presently, the contribution of the nursing workforce to overall population health is significantly less visible than physicians. This phenomenon reflects the inequity of power due to less visible measures of nursing influence, different levels of education, the perceived value of care as a job, historical hierarchies which continue to impact workplace structures. Nursing autonomy needs to be more explicit, supported and recognised. Without correction, health services will continue to transmit the invisibility of nursing healthcare from one generation of nurses to another.

## Data Availability

The sources of all the data have been described in detail in the “Study materials”. The formal permission to download and apply the data for non-commercial purpose is not required as per the protocol of the World Bank. All the data for this study are freely downloaded from the official website of the World Bank.
